# Efficient high-order suppression system for a metrology beamline

**DOI:** 10.1107/S1600577517016800

**Published:** 2018-01-01

**Authors:** A. Sokolov, M. G. Sertsu, A. Gaupp, M. Lüttecke, F. Schäfers

**Affiliations:** a Helmholtz Zentrum Berlin (BESSY-II), Albert-Einstein-Strasse 15, D-12489 Berlin, Germany

**Keywords:** reflectometer, higher orders, PGM beamline, at-wavelength metrology, XUV optical elements, diffraction gratings

## Abstract

High-quality metrology with synchrotron radiation requires in particular a very high spectral purity of the incident beam. This is usually achieved by a set of transmission filters with suitable absorption edges to suppress high-order radiation of the monochromator. The at-wavelength metrology station at a BESSY-II bending-magnet collimated plane-grating monochromator (c-PGM) beamline has recently commissioned a high-order suppression system (HiOS) based on four reflections from mirrors which can be inserted into the beam path. Two pairs of mirrors are aligned parallel so as not to disturb the original beam path and are rotated clockwise and counter-clockwise. Three sets of coatings are available for the different energy ranges and the incidence angle is freely tunable to find the optimum figure of merit for maximum suppression at maximum transmission for each photon energy required. Measured performance results of the HiOS for the EUV and XUV range are compared with simulations, and applications are discussed.

## Abstract 2

An efficient high-order suppression system has been developed and included in the optics beamline in BESSY-II synchrotron sources. The design of the instrument and its performance in suppressing high-order diffraction from the monochromator grating are described in this paper.

## 1. Introduction   

Extreme ultraviolet (EUV) and soft X-ray (XUV) optics are in great demand now more than ever due to significant developments in areas like EUV photolithography, X-ray/XUV gratings, space observation and tabletop XUV experiments (Barreaux *et al.*, 2017 ▸; Huang *et al.*, 2017 ▸; Müller *et al.*, 2017 ▸; Naujok *et al.*, 2016 ▸; Firsov *et al.*, 2013 ▸). Characterizations of the optics and performance tests need robust metrology techniques, which have been established as the ‘world’s standard for EUV and X-ray reflectance measurements’ in particular by the metrology laboratory of the German Physikalisch-Technische Bundesanstalt (PTB) (Krumrey *et al.*, 2014 ▸, and references therein) and by the Center for X-ray Optics (CXRO) reflectometer (Gullikson *et al.*, 2001 ▸).

In order to support our in-house developments on new concepts of XUV optical elements (Hafner *et al.*, 2015 ▸; Loechel *et al.*, 2013 ▸; Senf *et al.*, 2016 ▸; Chkhalo *et al.*, 2017 ▸; Braig *et al.*, 2017 ▸; Siewert *et al.*, 2018 ▸; Erko *et al.*, 2010 ▸), a versatile UHV reflectometer has been permanently installed as an end-station at the optics beamline at the BESSY-II synchrotron radiation source. The optical concept and design features of this facility have been described elsewhere (Sokolov *et al.*, 2016 ▸; Schäfers *et al.*, 2016 ▸). The design of the optics beamline as a collimated plane-grating monochromator mount (c-PGM) with a variable incidence angle pre-mirror has addressed all requirements for at-wavelength reflectometry: the beamline offers a broad photon energy range with energy resolution *E*/Δ*E* ∼ 1000–5000, flexible operation of the PGM in high-resolution, high-flux or high spectral purity mode, low divergence, sub-millimetre focus size, stray and scattered light suppression *via* aperture systems and the possibility to steer the polarization between linear (in-plane) and left/right elliptical (off-plane). The spectral purity of the beam, which is an essential property for at-wavelength metrology, is achieved either by inserting a set of absorption filters into the beam path (standard method) or by inserting a high-order suppression system (HiOS). Similar systems have been described elsewhere (*e.g.* Waki *et al.*, 1989 ▸; Frommherz *et al.*, 2010 ▸; Bulicke *et al.*, 1997 ▸).

The HiOS is based on four mirrors that are inserted into the beam path without net deflection of the beam trajectory. These two systems in the optics beamline provide a wide flexibility for light shaping upstream of the reflectometer; the properly aligned HiOS is by far more effective than the filter units (Schäfers *et al.*, 2016 ▸).

In this paper, we provide the design and working principles of the HiOS. Measured performance results in the UV and XUV ranges are compared with simulations, and applications in terms of diffraction efficiency measurements on gratings produced in-house are shown.

## 2. At-wavelength metrology facility   

### 2.1. Optics beamline   

**Figure 1:**
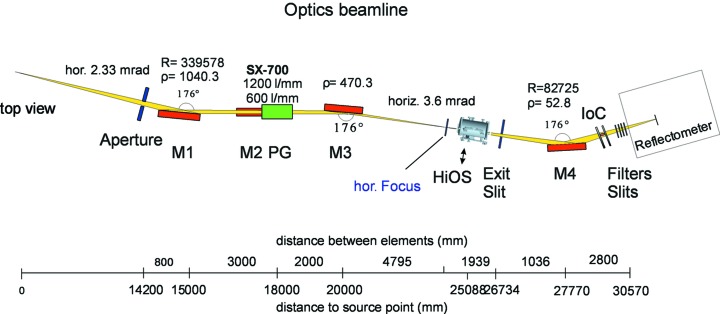
The at-wavelength metrology station at BESSY-II. Plane-grating monochromator beamline (c-PGM) and reflectometer at a bending-magnet port. The HiOS is indicated. IoC refers to ionization chamber.

The optics beamline at the BESSY-II dipole section DIP 1.1 aimed to establish an at-wavelength metrology facility for in-house research projects as well as for outside scientific user projects. The optical layout of the beamline is given in Fig. 1 ▸. Details may be found elsewhere (Sokolov *et al.*, 2014 ▸).

**Figure 2:**
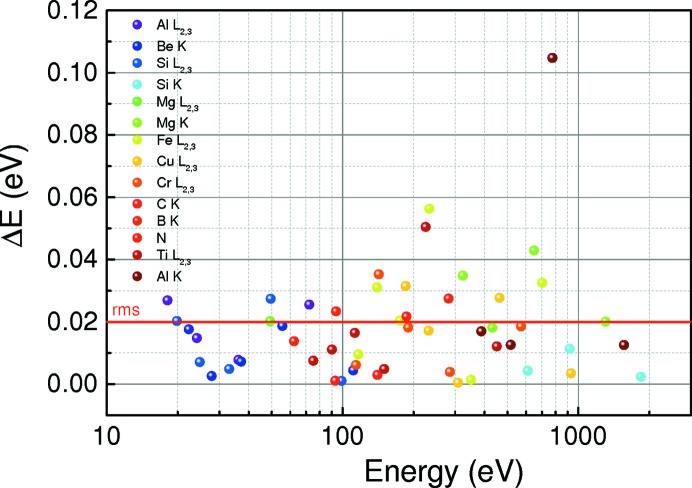
Energy calibration of the optics beamline. More than 50 reference energies are available *in situ* by use of absorption filters in first and all available higher orders.

The HiOS chamber is positioned between the horizontal and vertical foci created by the M1 and the M3 mirrors, respectively. The size of the beam here is about 4 × 5 mm (v × h). The M4 eventually focuses the beam onto the sample position inside the reflectometer chamber with a beam size of ∼0.36 × 0.2 mm (FWHM, v × h). The optics beamline also contains an ionization chamber mounted after the last mirror M4. This allows energy calibration of the monochromator and resolution tests by absorption spectroscopies of suitable gases. In addition, we carry out a refinement of the energy scale with the help of 12 *in situ* absorption filters which provide more than 50 structures (absorption edges in different diffraction orders from the monochromator) well distributed over the working energy range. To find precise energy positions for the mentioned structures, we routinely measure and calibrate them against well defined ionization lines of N_2_ in all available orders up to the fifth. The energy position for the first peak of the N_2_ 1*s* vibrational structure is at 400.77 eV (Sodhi & Brion, 1984 ▸). The reference energies, their energetic positions and accuracy for all available structures are summarized in Fig. 2 ▸. Energy accuracy of 0.02 eV can be obtained in most cases by this procedure.

**Figure 3:**
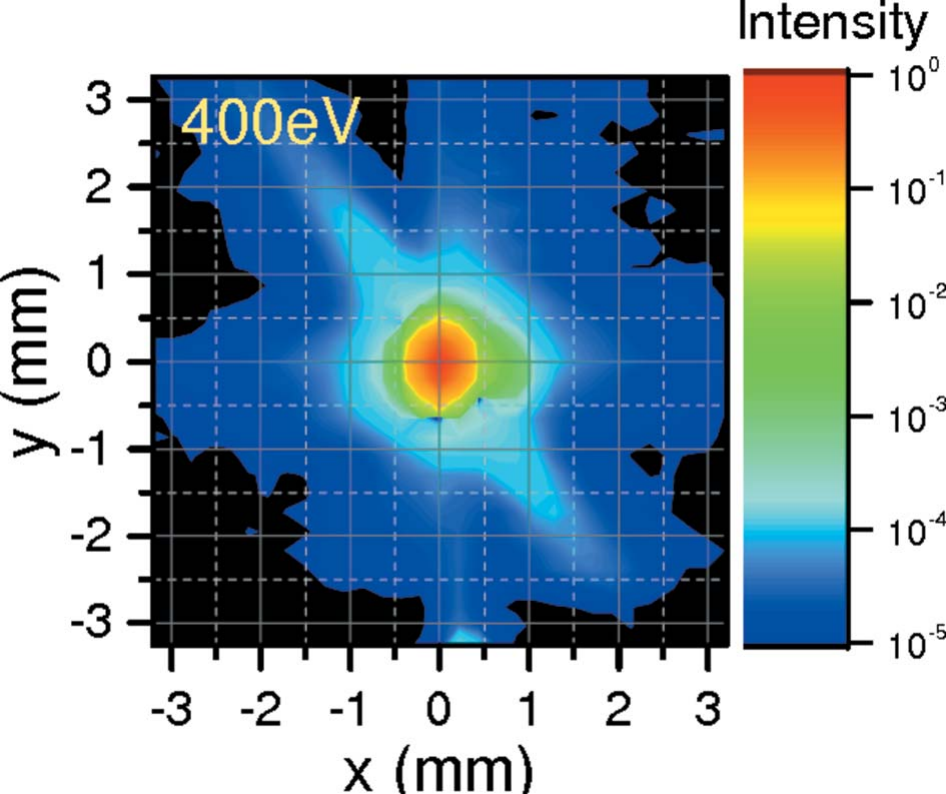
Two-dimensional mapping of the beam spot measured with the GaAsP photodiode detector in the reflectometer. FWHM of the spot size is 0.4 × 0.3 mm (v × h). Note the efficient stray light reduction by more than five orders of magnitude by suitable apertures and pinholes in the beamline.

A double set of pinholes and apertures installed in front of the reflectometer provides efficient stray light and scattered light suppression. Fig. 3 ▸ shows a typical beam spot captured with a pinhole detector of entrance aperture 0.1 mm situated 310 mm away from the focus point at the sample. An efficient stray light rejection in the order of 10^5^ is demonstrated.

### 2.2. Reflectometer   

**Figure 4:**
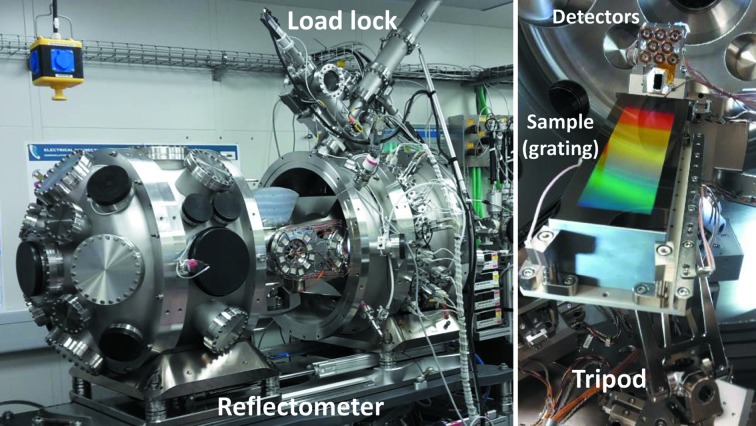
The UHV reflectometer with the recently installed motorized load-lock system on top (left). A real-size grating [190 × 60 × 45 mm (L × W × H)] is mounted on the sample holder (right).

The UHV reflectometer chamber is shown in Fig. 4 ▸. Details of the design and functionalities of the reflectometer are given by Eggenstein *et al.* (2014 ▸, 2016 ▸). The reflectometer possesses several features such as the possibility to mount real optical elements of size up to 360 mm in length and 4 kg in weight. It also owns a compactly designed UHV tripod system which allows sample alignment in six degrees of freedom and a two-dimensional sample mapping. The two goniometers for the sample holder and for the detector system allow measurement of reflectivity from 0° to 89° grazing angle and in-plane scattering in 4π. The possibility to rotate the whole system in the azimuth direction (*i.e.* around the beam direction) allows measurements in *s*- and *p*-polarization geometry. The detector system has an off-plane range from −4° to +4°. A load-lock system for rapid in-vacuum sample change has just been commissioned.

## 3. High-order suppressor   

**Figure 5:**
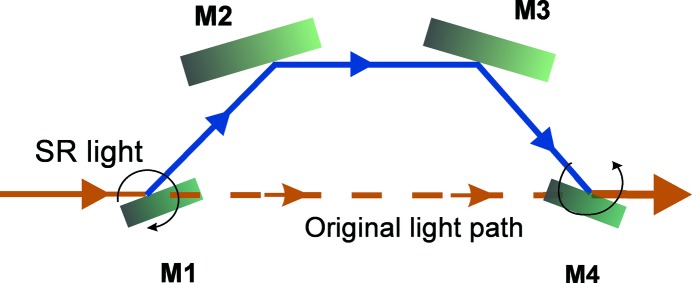
The positioning of HiOS mirrors. Light paths with and without HiOS are indicated.

The spectral purity is an essential merit for the at-wavelength measurements with the reflectometer. To achieve this, a HiOS was developed in-house to suppress high diffraction orders of the monochromator grating. The underlying optical principle of the HiOS design is that higher diffraction orders (*i.e.* higher photon energies) are suppressed (in contrast to the first-order energy) after consecutive reflections from four mirrors as shown in Fig. 5 ▸. By this approach the original trajectory of the beam is unaltered. A high-energy cut-off is freely selectable by tuning the incidence angle, due to a drop in reflectivity above the critical angle (depending on the coating material). The theoretical performance of the HiOS has been described previously (Sokolov *et al.*, 2016 ▸). According to this it should be possible to achieve a suppression efficiency of more than 10^4^ in the energy range of 20 to 700 eV while keeping the overall transmission up to 40%.

For photon-hungry experiments that are more intensity critical rather than dependent on spectral purity, the HiOS can be parked to a home position and the beam allowed to pass through. Alternatively absorption filters of choice can be used instead.

### 3.1. Instrument   

**Figure 6:**
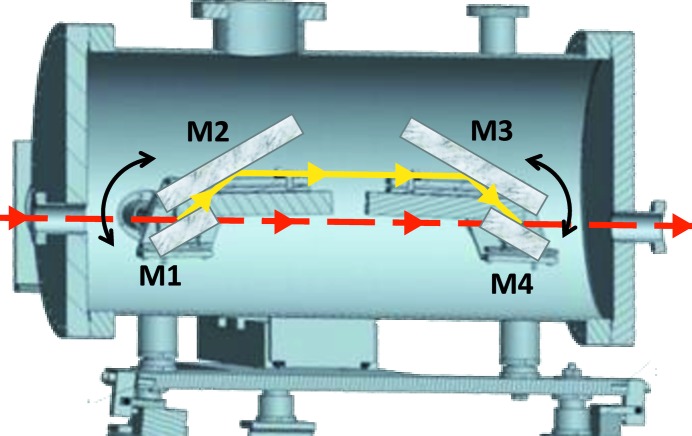
Schematic of the HiOS vacuum chamber and optical arrangement of the four mirrors 1, 2, 3 and 4.

A schematic of the HiOS chamber and arrangement of the four reflection mirrors M1–M4 is given in Fig. 6 ▸. The first two and the last two mirrors, aligned parallel to each other on common holders, are rotated by two goniometers in opposite directions to ensure there is no vertical offset of the reflected beam. Their rotation axes are in the centres of M1 and M4, respectively. The synchrotron radiation enters the HiOS vacuum chamber from the left and is diverted from its trajectory by M1. After reflection from the four mirrors consecutively, the beam is put back to its original trajectory by M4 just before it exits the chamber. When changing the incidence angle the intersection points of light onto M2 and M3 are travelling along these mirrors. Therefore the middle mirrors are longer and the vertical beam offset depends on the angle.

**Figure 7:**
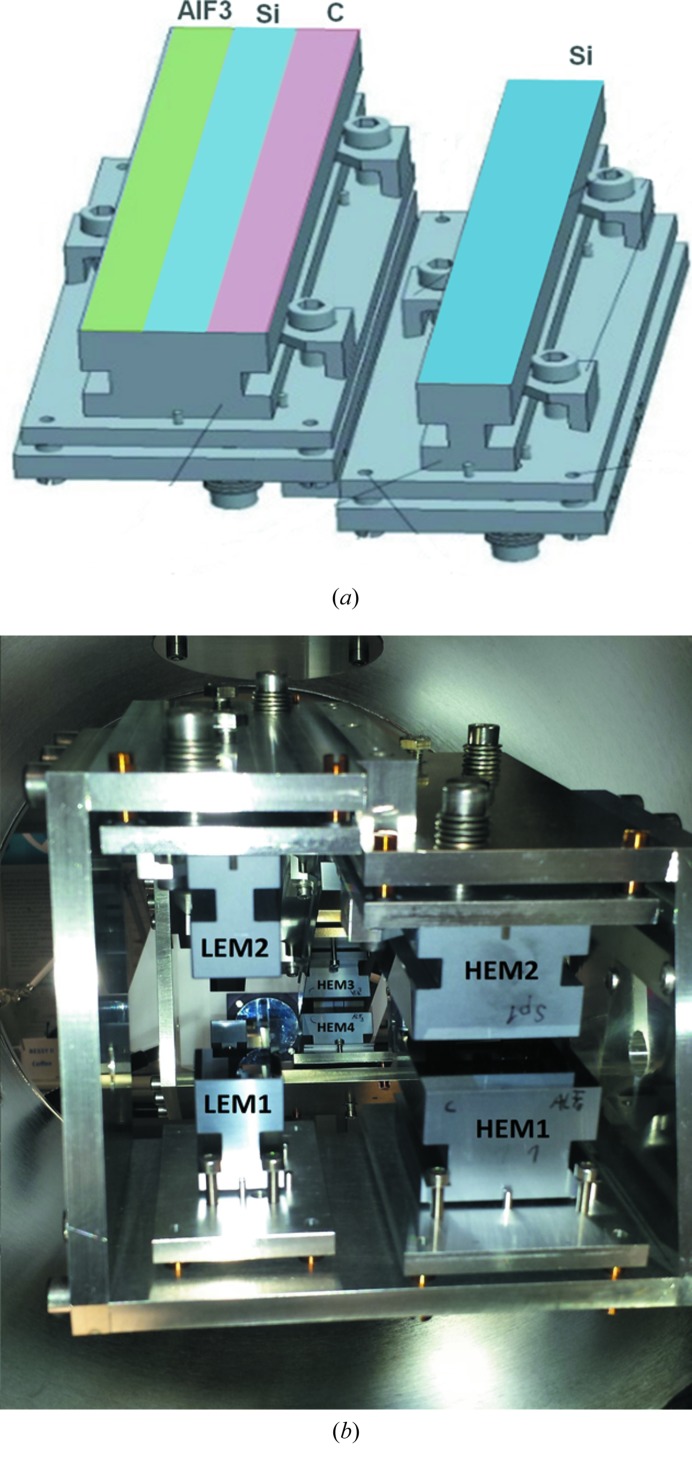
(*a*) M2 and M3 HiOS mirrors for the high- (left) and low- (right) energy range, respectively, coated with AlF_3_, Si (uncoated) and C (left) and uncoated (Si) (right). Mirrors #1 and #4 have identical coating stripes, but are shorter. (*b*) Photograph of the mirror box of the HiOS with low-energy (LEM1, 2) and high-energy mirrors (HEM1, 2).

It is apparent from the HiOS design that tuning of the incident angle and the use of different coating materials are sufficient to optimize suppression performance of the instrument in low- and high-energy ranges. In order to optimize HiOS for operation in a broad energy range (where the critical angle is changing from ∼2° to ∼35°) the chamber is equipped with two HiOS sets of four plane mirrors each. Both sets have different beam offsets between M1 and M2 (M3 and M4, respectively) for operation in different angular ranges. The first set, designed for low-energy (LEM) ranges from 10 to 60 eV, has four uncoated plane Si mirrors. The incident grazing angles can be tuned between 8° and 35°. The second set is meant for the high-energy range (HEM) at lower grazing-incidence angles. It contains three stripes with coatings of Si, C and AlF_3_ aimed at spanning the high-energy range from 50 to 1000 eV with a freedom in grazing angles from 2° to 15°. The left and right stripes in Fig. 7 ▸ are coatings of the upper mirrors (M2 and M3) for high-*E* and low-*E* ranges, respectively. M1 and M4 (not shown) are also made from the same materials but are shorter than M2 and M3.

The two sets of HiOS components with corresponding mirror coatings, working energy ranges and surface roughness characteristics are summarized in Table 1 ▸.

It is also worth mentioning that the HiOS motors are integrated into the EPICS environment of BESSY-II and are easily controlled *via* a *SPEC* command window. An auto-HiOS option in *SPEC* allows simultaneous scanning of photon energy and HiOS according to look-up tables for figure-of-merit operation, best transmission or best suppression operation.

### 3.2. Performance   

**Figure 8:**
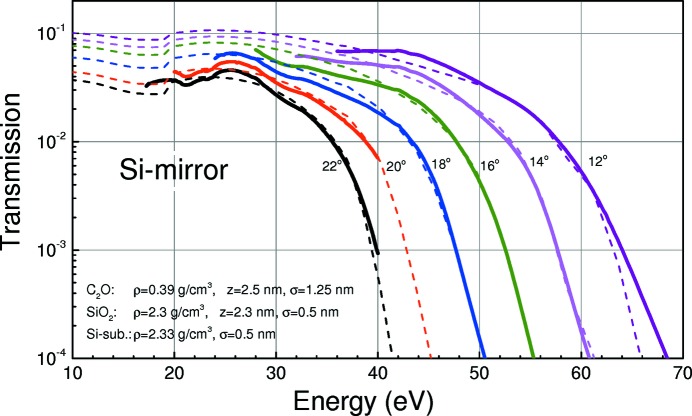
Measured (solid lines) and calculated (dashed lines) HiOS transmission for the (uncoated) Si substrates at different incidence angles. Calculation based on optical parameters obtained from fitting on test samples as indicated.

**Figure 9:**
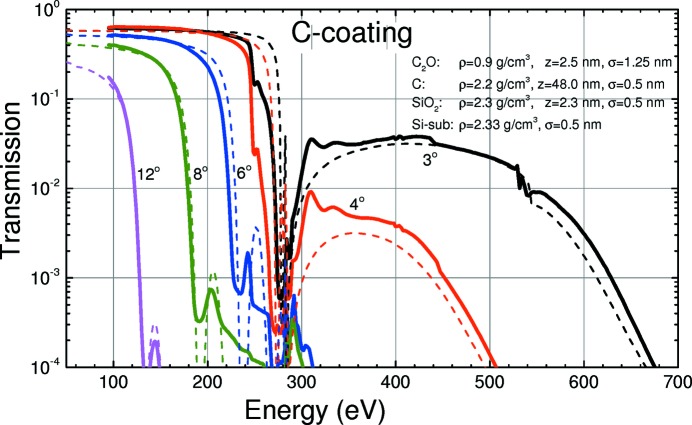
Measured (solid lines) and calculated (dashed lines) HiOS transmission for the C-coated substrates at different incidence angles. Calculation based on optical parameters obtained from fitting on test samples.

**Figure 10:**
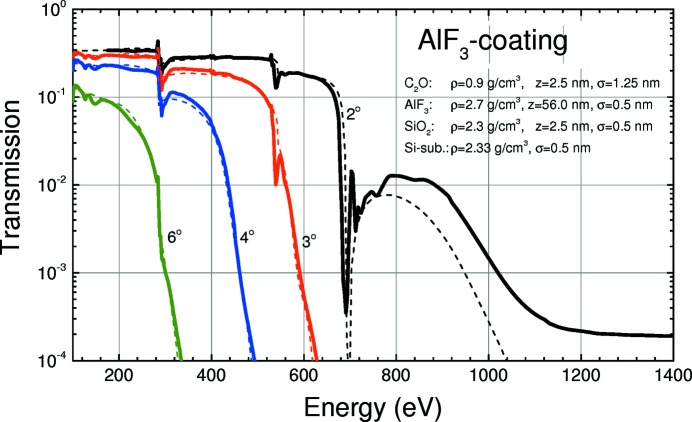
Measured (solid lines) and calculated (dashed lines) HiOS transmission for the AlF_3_ coating at different incidence angles. Calculation based on optical parameters obtained from fitting on test samples.

In this section the measured and the calculated performance of HiOS in terms of overall throughput and high-order suppression of the transmitted light are analysed. Parts of the results are shown in Figs. 8 ▸, 9 ▸ and 10 ▸. They show the measured and calculated (see below) HiOS transmission for the Si substrate (Fig. 8 ▸), the C coating (Fig. 9 ▸) and the AlF_3_ coating (Fig. 10 ▸) for different grazing-incidence angles.

For the simulation of the calculated curves (dashed lines) in Figs. 8 ▸, 9 ▸ and 10 ▸, reflectivity measurements and fit simulations on test samples were performed previously (not shown). From these investigations an optical model was developed for each coating. This model takes into account a top contamination layer and a buried interface layer of SiO_2_ between the Si substrate and the coating, assuming uniform density. Thickness, roughness and density for each layer were obtained from fitting to angular reflectivity data at several photon energies. Parameters were optimized in such a way to give best agreement in a broad energy range.

Overall agreement between the calculated and measured data in Figs. 8 ▸, 9 ▸ and 10 ▸ is good. This is even true for the vicinity of the C and O *K* absorption edges using the AlF_3_ coating (Fig. 10 ▸). The C edge on mirrors with a C coating cannot be fitted satisfactorily because of a lack of reliable optical constants. However, this is not a good HiOS coating for that particular absorption edge anyway.

As the figures show, the availability of three different coated areas and a smooth variation of incidence angles give us a large flexibility to select optimum HiOS operation parameters for each individual energy. In order to get the best selection in this parameter space we developed a figure-of-merit function (FoM) as:
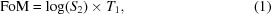


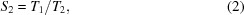
where *T*_1_ is the transmission of first order, *T*_2_ the transmission of second order and *S*_2_ suppression of the second order. Both values *T*_1_ and *T*_2_ were obtained from the optical models described in Figs. 8 ▸–10 ▸, and *T*_2_ can be easily calculated as *T*_1_ at double energy. For definition of higher-order suppression we take into account second-order suppression only, since usually higher orders have even lower reflectivity/transmission. In order to have a good balance between suppression and transmission we use the logarithm of *S*_2_ since we are interested in having a suppression of some orders of magnitude at still reasonable transmission.

**Figure 11:**
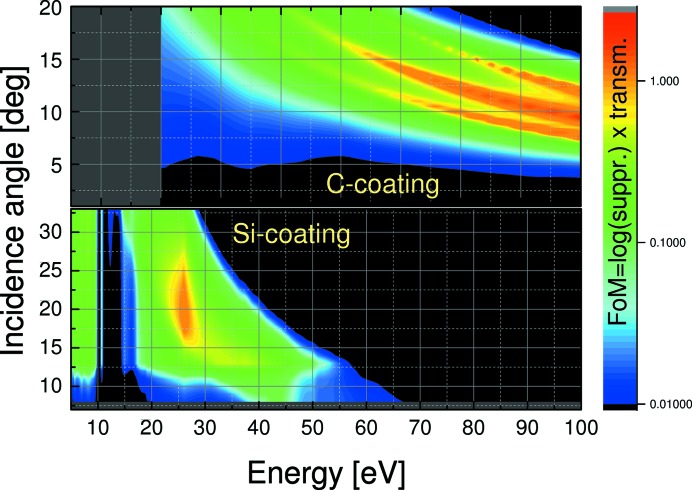
FoM mapping of HiOS incidence angle *versus* photon energy for low energies and Si and C coatings.

The two-dimensional maps of this FoM as a function of incidence angle and photon energy for all coatings are presented in Figs. 11 ▸ and 12 ▸. These figures were obtained as simulations with *IMD* software (Windt, 1998 ▸) using optical models discussed above (Figs. 8 ▸, 9 ▸ and 10 ▸).

We limited the minimum FoM value to 0.01 assuming that below this value either the suppression or transmission would be too low. Thus the black regions at low angles represent poor suppression, while the black regions at large angles represent too low first-order transmission. The optimum working ranges of the coatings in low and high energy extracted from Figs. 11 ▸ and 12 ▸ are summarized in Table 2 ▸.

The *SPEC* data acquisition program of our facility allows operation of the HiOS during an energy scan either on the maximum FoM curve with continuous movement of the HiOS motors for incidence angle and coating selection, or, in cases where beam stability requirements are high, the energy range can be split into sections with different but fixed settings of the HiOS. For this operation look-up tables other than the FoM table are also available, depending on whether higher priority is given to transmission (smaller incidence angles) or suppression (larger incidence angles).

**Figure 13:**
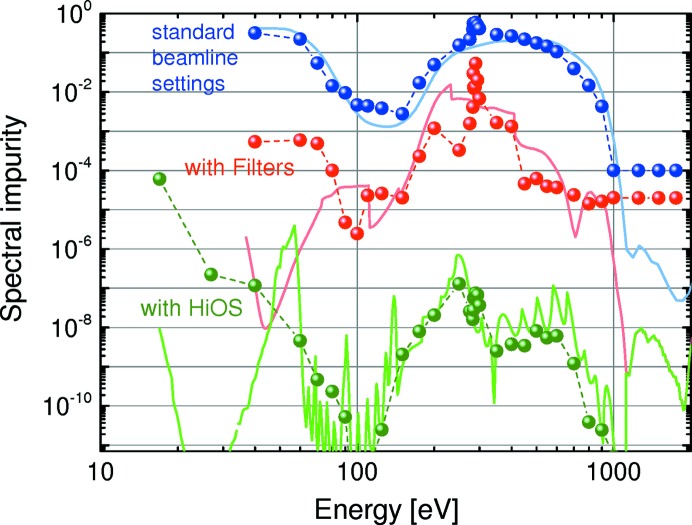
Measured (points) and calculated (solid lines) spectral impurity of the optics beamline in standard PGM operation mode (*c* = 2.25) (blue), with absorption filters (red) and with HiOS (green).

The versatility of HiOS in terms of available optical coatings, tailoring of the incidence angles and working energy ranges to optimize the transmission has been described above. Hereafter, we explain the suppression performance of the HiOS in comparison with the absorption filters. Fig. 13 ▸ summarizes all results in terms of spectral impurity, which is the ratio of the sum of all higher-order intensity to first-order intensity. All data were obtained by measurements of the dispersion pattern of test gratings in the reflectometer. The figure shows the measured and calculated spectral impurity of the optics beamline (i) in standard PGM operation mode (*c* = 2.25, 1200 lines mm^−1^ grating), (ii) with absorption filters and variable *c* values and (iii) with the HiOS. The HiOS data were obtained partly by simulation of the suppression values [based on the transmission curves (Figs. 8 ▸, 9 ▸ and 10 ▸)], since the impurity values are too small to be measured due to the limited dynamic range of the system.

All calculations of Fig. 13 ▸ were carried out with the *REFLEC* program (Schäfers & Krumrey, 1996 ▸). The beamline transmission including all optical elements was calculated in every available diffraction order. The agreement between measured points and calculation for case (i) is excellent, except for the C *K*-edge region (C contamination was not taken into account in calculations) and for the high-energy range above 1000 eV, where the dynamic range limits the measuring accuracy to >10^−4^. This good agreement gives a sort of error bar for the Fig. 13 ▸ data and solidifies this chosen evaluation procedure for the spectral impurity.

In case (ii) the steps in the calculation represent the filter change, while the low- and high-energy ranges again have limited dynamic range. Again, contamination has not been taken into account in the calculations.

The simulated HiOS curve [case (iii)] has significant oscillations in the medium-energy range; this is, however, an artefact due to the selected step sizes in angular positioning and coating of the HiOS. In a continuous HiOS angle change with energy, the oscillations would vanish and approach the experimental data. Therefore the overall agreement with experimental data is very good and, in most of the energy range, better than experimentally measurable anyway due to the limited dynamic range. This is also the reason for the theoretical minimum at 35 eV, which is hardly indicated in the experimental data points. Above 1000 eV all three curves represent standard beamline operation without filters or HiOS; however, the *c* factor is different [2.25 for case (i) and 1.5 for cases (ii) and (iii)].

Obviously the spectral purity for operation with HiOS is orders of magnitude higher than with filters. One of the reasons for this is clear, since, in general, filters suppress only second order. Moreover the HiOS extends the working range easily to lower energies, since there are no good absorption filters below 37 eV. In this range alternative solutions such as rare gas filters have been suggested (Gottwald *et al.*, 2010 ▸).

**Figure 14:**
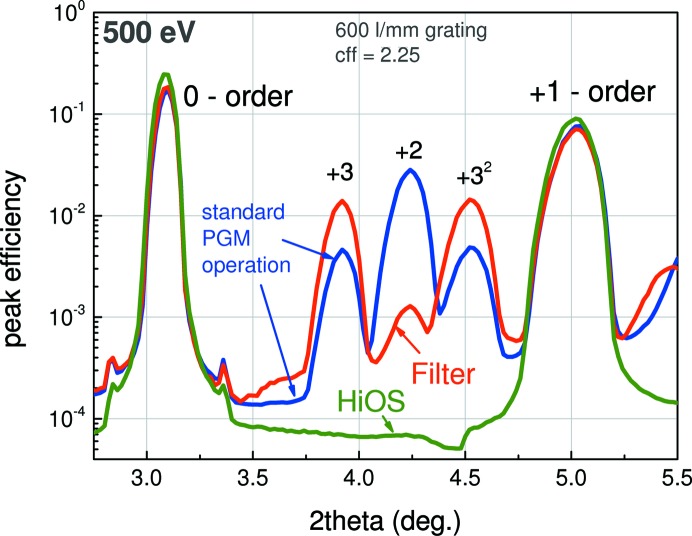
Diffraction efficiency of a blazed 600 lines mm^−1^ grating measured in standard PGM operation mode (*c* = 2.25). Colour code as in Fig. 13 ▸. Using an Fe (750 nm) filter (red curve) only the second order can be suppressed; the HiOS (operated with AlF_3_-coated mirrors at 2.5°) (green curve) suppresses all higher orders.

To prove the reliability of the presented HiOS performance in suppressing high orders we measured the diffraction patterns of a blazed grating (600 lines mm^−1^) mounted inside the reflectometer in a wide energy range between 17 and 1000 eV. As a convincing example of the benefit of HiOS one of these results is presented in Fig. 14 ▸. The grating was measured in three different configurations of the beamline: (i) standard PGM operation (*c* = 2.25), (ii) same PGM configuration using a suitable absorption filter (750 nm Fe), (iii) same PGM configuration using the HiOS (AlF_3_ coating at 2.5°). The dispersion curve between zero (0) and first-order (+1) diffraction was scanned with the 2θ detector. The second (+2) and third order (+3) from the monochromator together with this order, diffracted in second order (+3^2^) by the grating under test, show up. It is clearly seen that HiOS suppresses all higher orders very efficiently, while filters are good at suppressing the second-order diffraction only.

## 4. Conclusions   

We have described a novel high-order suppression system (HiOS) installed in the optics beamline of BESSY-II which is coupled permanently to a UHV reflectometer in a clean-room hutch. The motivation for the development of HiOS originates from the need to suppress high-order radiation from the monochromator grating very efficiently to allow high-precision at-wavelength metrology with high spectral purity. The HiOS, besides the absorption filters in the filter-slit unit chamber (FSU), bestows additional flexibility for this purpose. The suppression capability of HiOS becomes even better at higher orders unlike the absorption filters which show optimized suppression only for the second order.

The HiOS chamber contains several mirrors with ample freedom to tailor the incidence angles and material coatings. HiOS, unlike the absorption filters, executes efficient suppression of all high orders with typical optical transmission of about 0.1% to 40% depending on the user’s preference for higher transmission or higher suppression. A reasonable compromise between both options is the operation at the best figure-of-merit coating and angle.

Measurements and simulations described in this paper confirm superior spectral purity performance of the HiOS in a wide energy range from UV to soft X-rays. As a result, measurements of the diffraction efficiency of gratings, narrow band reflectivity of multilayers and other applications can be performed with very high accuracy.

## Figures and Tables

**Figure 12:**
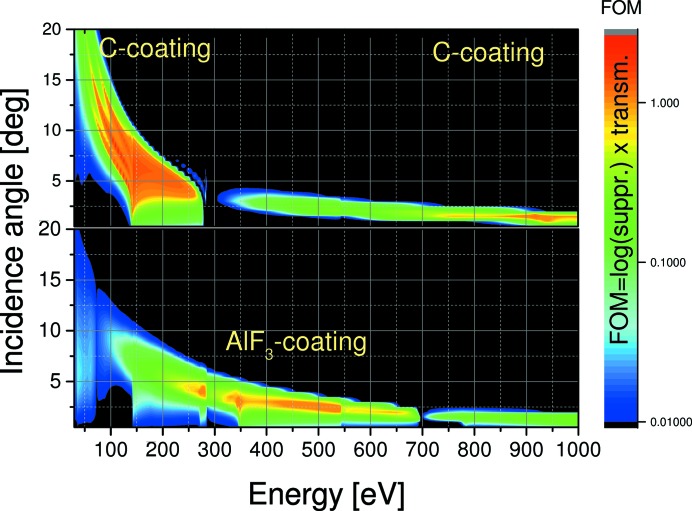
FoM mapping of HiOS incidence angle *versus* photon energy for high energies and AlF_3_ and C coatings.

**Table 1:** Parameters of the HiOS mirrors and their alignment with respect to each other Surface roughness and slope error were measured with white light interferometry and slope-measuring deflectometry (Siewert *et al.*, 2014 ▸). RMS = root mean square.

Parameters	HiOS #1 (low *E*)	HiOS #2 (high *E*)
Energy range	10–60 eV	50–1000 eV
Angular range	8–35°	2–15°
Size of mirror 1, 4	20 × 20 mm	40 × 40 mm
Size of mirror 2, 3	180 × 20 mm	180 × 40 mm
Vertical acceptance	2.8–18. 8 mm	1.4–8.3 mm
Mirror separation	22 mm	5 mm
Material	Si	Si
Surface geometry	Plane	Plane
Surface roughness	<0.5 nm RMS	<0.5 nm RMS
Slope error	<0.3 arcsec RMS	<0.3 arcsec RMS
Coating stripe 1		AlF_3_ (50 nm)
Coating stripe 2	Si (no coating)	Si (no coating)
Coating stripe 3		C (50 nm)
Alignment (H/V)	0/20 µm	0/70 µm
Alignment (H/V)	100/−20 µrad	82/8 µrad
Energy shift	<30 meV @ 100 eV	<1 eV @ 1000 eV

**Table 2:** Optimum energy and angular working ranges of Si mirror and coatings available in HiOS

Coating material	Energy range	Angular range
Si mirror	<55 eV	35–12°
C coating	50–240 eV	18–5°
AlF_3_ coating	230–640 eV	5–2.5°
C coating	>630 eV	<2.5°
